# Co-Release of Cytarabine and Polyphenol-Rich Extract from Polycaprolactone Microparticles Towards Leukemia Therapy

**DOI:** 10.3390/polym18030394

**Published:** 2026-02-02

**Authors:** Jenifer Leyva Castro, Laura A. de la Rosa, Emilio Álvarez Parrilla, Imelda Olivas Armendáriz, Jazmín Cristina Stevens Barrón, Christian Chapa González

**Affiliations:** 1 Laboratorio de Nanomedicina, Instituto de Ingeniería y Tecnología, Universidad Autónoma de Ciudad Juárez, Ciudad Juárez 32310, Mexico; 2 Departamento de Ciencias Químico-Biológicas, Instituto de Ciencias Biomédicas, Universidad Autónoma de Ciudad Juárez, Ciudad Juárez 32310, Mexico; ldelaros@uacj.mx (L.A.d.l.R.); ealvarez@uacj.mx (E.Á.P.); 3 Departamento de Física y Matemáticas, Instituto de Ingeniería y Tecnología, Universidad Autónoma de Ciudad Juárez, Ciudad Juárez 32310, Mexico; iolivas@uacj.mx; 4 Departamento de Ciencias Veterinarias, Instituto de Ciencias Biomédicas, Universidad Autónoma de Ciudad Juárez, Ciudad Juárez 32310, Mexico

**Keywords:** polymer, cancer, chemotherapy, cytarabine, polyphenol, extract, carrier, emulsion, delivery, particle

## Abstract

Polymer-based drug delivery systems offer robust opportunities to improve chemotherapy performance while mitigating systemic toxicity, a critical challenge in leukemia treatment. In this study, poly(ε-caprolactone) (PCL) microparticles were developed as carriers for the co-delivery of cytarabine (ARA-C), a frontline antileukemic agent, and a pecan-derived polyphenolic extract (PRE) as a complementary bioactive component. Microparticles were prepared by a double emulsion solvent evaporation method and formulated with varying drug and extract loadings. The systems were characterized in terms of morphology, particle size, colloidal properties, encapsulation efficiency, and chemical composition using optical microscopy, scanning electron microscopy, dynamic light scattering, zeta potential analysis, UV–Vis spectroscopy, Folin–Ciocalteu assay, and FTIR spectroscopy. *In vitro* release studies revealed sustained and formulation-dependent release profiles for both ARA-C and PRE, which were successfully fitted to kinetic models, indicating diffusion- and matrix-controlled release mechanisms. Additionally, preliminary cell viability assays using fibroblasts supported the cytocompatibility of the formulations. The results support the use of PCL-based microparticles as reproducible polymeric systems for the co-encapsulation and controlled release of cytarabine and polyphenol-rich extracts, contributing to the development of combination delivery approaches relevant to leukemia treatment.

## 1. Introduction

Cancer is a major global health challenge, with leukemia being one of the most common malignancies in children [1]. Acute lymphoblastic leukemia (ALL)—a neoplastic proliferation of lymphoid progenitors—is the predominant childhood cancer, comprising roughly 25–30% of pediatric malignancies [2]. Conventional ALL therapy relies heavily on multi-agent chemotherapy [3]. Among these agents, cytarabine (ARA-C) is a deoxycytidine analog widely used in protocols for both ALL and other leukemias [4,5,6,7,8]. Inside tumor cells, ARA-C is phosphorylated to its active triphosphate form, which competitively inhibits DNA polymerase and blocks DNA elongation, thereby inducing apoptosis [6,9,10]. However, ARA-C therapy is limited by poor pharmacokinetics and toxicity: the drug has a very short plasma half-life [11], low stability [12] and bioavailability [13], and dose-dependent side effects such as myelosuppression and neurotoxicity [14]. These drawbacks often require continuous infusions and supportive interventions, motivating the development of improved delivery systems.

Complementary approaches to mitigate these issues include combining chemotherapy with antioxidants [15]. Dietary polyphenols have attracted attention as adjuncts in leukemia therapy due to their intrinsic anticancer and antioxidant activities [16]. Polyphenols can influence oncogenic pathways, arrest the cell cycle, and promote apoptosis in malignant cells, while also scavenging reactive oxygen species (ROS) that are often elevated in cancer or induced by treatment [17,18,19,20].

The pecan nut (*Carya illinoinensis*) is a rich source of bioactive polyphenols and tocopherols whose composition has been well characterized [21,22,23,24]. Pecans contain high levels of flavonoids, ellagitannins (precursors to ellagic acid), and gamma-tocopherol, which is a potent vitamin E form [25,26,27,28]. Upon ingestion, ellagitannins are metabolized to ellagic acid and urolithins, which enhance antioxidant enzyme activity and upregulate pro-apoptotic pathways [29]. Thus, pecan nut polyphenolic extract (PRE) has strong antioxidant capacity and has been investigated for anti-inflammatory and anticancer potential [30,31]. Co-delivering PRE with chemotherapy could synergistically attenuate ROS overproduction, while maintaining or enhancing anticancer efficacy.

Polymeric particles are promising platforms for co-delivery of drug combinations. Polyesters such as poly(ε-caprolactone) (PCL) can form microparticles or nanoparticles that encapsulate hydrophilic and hydrophobic agents [32,33,34,35]. These carriers can modulate drug release kinetics and improve stability. The solvent evaporation double emulsion method is a well-established technique to fabricate polymeric microparticles with encapsulated agents [36,37,38,39,40]. In this approach, an aqueous solution of hydrophilic actives, like ARA-C and various polyphenols, is emulsified into a volatile organic solution of polymer, for example, PCL in chloroform, and that primary emulsion is further emulsified into an external aqueous surfactant phase, such as PVA [41,42]. Upon solvent removal, polymeric particles form, entrapping the agents.

The objective of this study was to design and evaluate a functional polymer-based co-delivery system capable of simultaneously encapsulating a conventional antileukemic drug and a natural antioxidant source. In this work, poly(ε-caprolactone) (PCL) microparticles co-loaded with cytarabine (ARA-C) and a pecan nut-derived polyphenol-rich extract (PRE) was developed using a water–oil–water double emulsion solvent evaporation approach. PCL was selected as the matrix polymer because of its established biocompatibility, ease of fabrication by solvent evaporation methods, and slow hydrolytic degradation, which enables prolonged drug release profiles suitable for sustained delivery applications. PCL’s processing versatility and safety profile for biomedical use have been widely reviewed [43,44,45]. This work provides an analysis of how drug loading influences encapsulation efficiency, colloidal stability, and release behavior, contributing mechanistic insight into functional polymer design for combination drug delivery. Furthermore, a preliminary *in vitro* cellular viability assessment using fibroblasts was conducted to evaluate the cytocompatibility of the developed microparticles. This study contributes new experimental evidence supporting the use of functional polymeric systems for the rational co-delivery of chemotherapeutic agents and natural antioxidants.

## 2. Materials and Methods

### 2.1. Materials

Poly(ε-caprolactone) (PCL; Mw ~80 kDa), poly(vinyl alcohol) (PVA; 87–89% hydrolyzed, Mw 30–70 kDa), phosphate-buffered saline (PBS) tablets, and MTT (3-(4,5-Dimethylthiazol-2-yl)-2,5-Diphenyltetrazolium Bromide) were procured from Sigma-Aldrich (St. Louis, MO, USA). Cytarabine (ARA-C; Zurich) was acquired from Araujo Pharmacy (San Pedro Garza García, N.L., Mexico), a pharmaceutical supplier. Organic solvents—methanol (≥99.8%); n-hexane (95%, analytical grade) and chloroform (≥99.8%)—were obtained from suppliers Licomex (Mexico City, CDMX, Mexico) and CTR (Monterrey, N.L., Mexico), respectively. Folin–Ciocalteu reagent was supplied by Merck (Darmstadt, Germany).

### 2.2. Extraction of Polyphenol-Rich Extract (PRE)

PRE was prepared using a standardized extraction protocol, previously reported [21]. Pecan nuts (*Carya illinoinensis*) were obtained from a commercial orchard in Cuauhtémoc, Chihuahua, Mexico. The kernels, mechanically shelled at the production site, were supplied as fragments and stored in opaque metal containers until processing. For the extraction of phenolic compounds, 20 g of shelled pecans were used, mechanically ground to obtain a fine powder. This powder was defatted by two successive extractions with n-hexane (10 mL g^−1^) assisted by sonication at 40 °C for 30 min, followed by centrifugation at 3500 rpm and 4 °C for 15 min. The defatted residue was dried at 50 °C for 24 h. Subsequently, the phenols were extracted from the dry powder using 30 mL of 80% (*v*/*v*) aqueous methanol by sonication at 40 °C for 30 min. The extract obtained, after a centrifugation step under identical conditions, was concentrated by rotary evaporation at 50–55 °C and 175 mbar. The final concentrate was freeze-dried, yielding a dry powder that was stored under vacuum until analysis.

### 2.3. Calibration Curves

Separate calibration curves were established for ARA-C and PRE. ARA-C calibration standards were prepared by serial dilution of the injectable ARA-C stock in phosphate-buffered saline (PBS, pH 7.4). For ARA-C, standard solutions of the injectable formulation, covering the range 0.5–50 µg/mL, were scanned by UV-Vis and found to have peak absorbance at 285 nm. Using this wavelength, absorbance was measured for each standard concentration. For PRE, a 10 mg/mL stock, dissolved in 80% (*v*/*v*) methanol–water, was diluted in the range 0.30–10.0 mg/mL and reacted with Folin–Ciocalteu reagent; absorbance at 765 nm gave a linear calibration for phenolic content. All calibration curves were linear over the indicated ranges (R^2^ > 0.99). These curves were used to quantify ARA-C and total polyphenols in encapsulation and release studies.

### 2.4. Microparticle Preparation (Double Emulsion)

PCL microparticles were prepared by a modified W/O/W solvent evaporation method, described elsewhere, with modifications [46]. The primary W/O emulsion was produced by probe ultrasonication using a 20 kHz probe sonicator (Ultrasonic Processor, ZL-800) at 40% amplitude with a continuous regime for 10 min. Immediately after, the primary emulsion was poured into the PVA aqueous phase and the secondary W/O/W emulsion was formed by subjecting the phases to a second sonication with the same parameters. Chloroform was evaporated under stirring at 400 rpm using a heated stirring plate (MS-H280-Pro, DLab Scientific Co., Johor Darul Ta’zim, Malaysia), causing PCL precipitation and particle formation. After solvent evaporation, the resulting suspensions were centrifuged (3000 rpm, 5 min) to separate the microparticles from the supernatant. This step was used to remove excess stabilizer (PVA) and unencapsulated compounds prior to encapsulation efficiency and release analyses. Microparticles were otherwise characterized and stored as aqueous suspensions. For SEM analysis, aliquots were dried prior to imaging.

Samples were coded sequentially as M1–M8, where “M” denotes microemulsion formulation and each number identifies a unique composition. Each formulation was prepared as an independent batch and labeled accordingly; three independent batches per formulation were produced for physicochemical characterization and release studies. Table 1 summarizes the composition and key preparation parameters for each formulation. All formulation codes are referenced throughout the Section 3 to indicate the specific sample evaluated.

### 2.5. Microparticle Characterization

Immediately after formation (day 0) and after 21 days storage, particles were observed by optical microscopy (phase contrast, Motic BA210E, Xiamen, China) to assess morphology and size. The wet suspensions were drop-cast on slides for imaging. Particle diameters were estimated with ImageJ (version 1.54g) from representative micrographs. Colloidal stability of each formulation was monitored by turbidimetry: aliquots of each suspension were measured at 600 nm in a microplate reader (Varioskan LUX, ThermoScientific, Waltham, MA, USA) every 5 min for 12 h on days 0, 7, 14, 21, and 28. These kinetic transmittance profiles indicate phase separation or flocculation over time.

Surface morphology was examined by scanning electron microscopy (SEM, Hitachi SU5000, Minato-ku, Tokyo, Japan). Particles from selected formulations (M1, M6, M7, M8) were collected by centrifugation, mounted on carbon tape, sputter-coated, and imaged at various magnifications. Size distribution and zeta potential of particles were measured by dynamic light scattering (DLS). Samples of M1, M6, M7, and M8 were diluted 100-fold in PBS 1× pH = 7.4 and analyzed in an instrument (Nanotrac Wave II, Microtrac, Haan, Nordrhein-Westfalen, Germany). The average hydrodynamic diameter and surface potential (zeta) were recorded for each. Attenuated total reflectance Fourier transform infrared spectroscopy (ATR-FTIR) was used to determine the functional groups of the different formulations using an infrared spectrophotometer (Nicolet iS10, Thermo Scientific, Waltham, MA, USA).

### 2.6. Encapsulation Efficiency (EE)

Encapsulation efficiency was determined using an indirect method based on quantification of the non-encapsulated fraction in the supernatant following particle formation. Briefly, aliquots of each formulation were centrifuged at 3000 rpm for 5 min to separate the particle pellet from the supernatant. The combined supernatants (initial and wash fractions) were analyzed for free ARA-C by UV–Vis spectroscopy (absorbance at 285 nm) and for PRE by Folin–Ciocalteu assay (absorbance at 765 nm). Encapsulation efficiency (%) was calculated as follows:

$$EE\;(\%)=\frac{m_{initial}-m_{free}}{m_{initial}}\times 100$$

where *m_initial_* is the total mass of compound added during formulation, and *m_free_* is the mass quantified in the supernatants. The indirect supernatant method is widely used for polymeric microparticles and nanoparticles, particularly for hydrophilic compounds [47,48,49].

### 2.7. In Vitro Release Studies

Drug release was studied using a dialysis membrane method. Approximately 5 mL of microemulsion was placed inside a cellulose membrane (MWCO 1000 Da) sealed in a plastic vial, immersed in 10 mL of phosphate-buffered saline (PBS, pH 7.4) at 37 °C. At specified times (1, 3, 5, 10, 20, 30, 40, 50, 60, 80, 100, 120, 150, 180, 210, 240, 360, 1440, 2880 min), aliquots (25 µL) were removed from the PBS and analyzed by UV-Vis microplate reader (Varioskan LUX, ThermoScientific) (ARA-C at 285 nm; PRE at 765 nm after Folin reaction). Concentrations were calculated via the calibration curves. Each formulation was tested in triplicate. Cumulative release (%) was plotted versus time.

Release data were fit to kinetic models (zero-order, first-order, Higuchi, Hixson–Crowell, and Korsmeyer–Peppas) using regression to determine the best-fitting release mechanism. Statistical analysis (one-way ANOVA) was performed on release percentages at 48 h for formulations with differing ARA-C loads (50, 5, 0.5 mg/mL) to test the effect of drug concentration. Data normality was checked, and log transformation was applied as needed. Tukey’s test was used for pairwise comparisons.

### 2.8. Cell Viability Assay

Cell viability was evaluated using the MTT assay in murine fibroblast subcultures of Balb/c origin. These cells correspond to an established fibroblast culture previously isolated from Balb/c mice and characterized under approved ethics protocols, as reported by Martinez-Osuna et al. [50]. The fibroblast cells were obtained through an institutional collaboration with the Biomaterials and Cell Culture Laboratory, Universidad Autónoma de Ciudad Juárez, and were used in the present study as subcultured cells, rather than primary cultures. Cells were cultured in Dulbecco’s Modified Eagle Medium (DMEM) supplemented with heat-inactivated fetal bovine serum and antibiotics and incubated at 37 °C in a humidified atmosphere containing 5% CO_2_. Fibroblasts were seeded in 96-well plates and exposed to PCL-based microparticle formulations, while PRE-treated cells were used as controls. After incubation periods of 24 and 48 h, MTT solution (5 mg/mL) was added to each well and incubated for an additional hour. Formazan crystals were solubilized in dimethyl sulfoxide (DMSO), and absorbance was measured at 570 nm using a microplate spectrophotometer. Cell viability was expressed as a percentage relative to controls.

## 3. Results and Discussion

**Figure 1:**
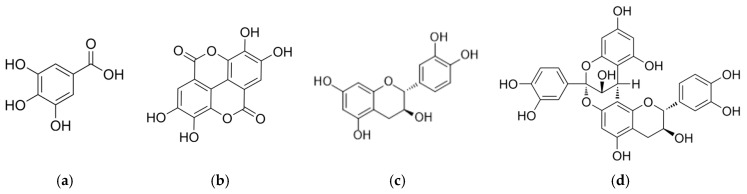
Representative chemical structures of principal phenolic constituents commonly found in pecan nut extracts and expected in PRE: (**a**) gallic acid; (**b**) ellagic acid; (**c**) (+)-catechin; and (**d**) proanthocyanidin dimer (representative oligomer).

Twenty grams of defatted pecan kernel powder were processed to obtain the polyphenol-rich extract (PRE). After sonication-assisted solvent extraction, concentration, and freeze-drying, the dry PRE mass was recorded. The final mass of freeze-dried PRE obtained from 20.0 g of starting defatted powder was 1.750 g, corresponding to an extraction yield of 8.75% *w*/*w*. The PRE is a complex extract enriched in low-molecular-weight phenolics and oligomeric tannins typically reported for *Carya illinoinensis*. Based on published phytochemical analyses, the major small-molecule constituents expected in PRE include gallic acid, ellagic acid, (+)-catechin and (−)-epicatechin, together with proanthocyanidin oligomers and other phenolic acids (Figure 1) [51,52,53]. Total phenolic content was quantified by Folin–Ciocalteu assay.

**Figure 2:**
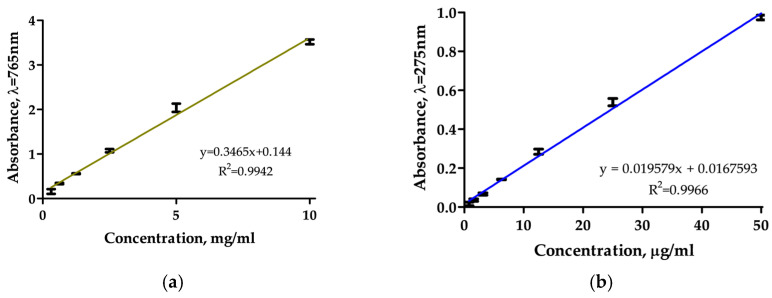
Calibration curves used in this study: (**a**) PRE calibration by Folin–Ciocalteu (absorbance at 765 nm). (**b**) ARA-C calibration in PBS (pH 7.4), absorbance at 285 nm. Regression displayed.

The PRE calibration with Folin–Ciocalteu method at 765 nm gave a linear fit (Figure 2a) described by y = 0.3465x + 0.144, R^2^ = 0.9942, which enables quantification of total phenols in assays. The ARA-C absorbance spectrum showed a λ_max_ at 285 nm, yielding a linear calibration (R^2^ = 0.9966) described by y = 0.0196x + 0.0168 (Figure 2b).

**Figure 3:**
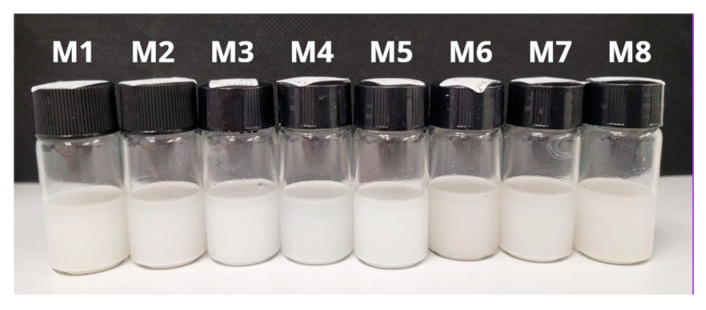
Photographs of formulations M1 to M8 show a homogeneous milky appearance, with the shade varying depending on the composition.

**Figure 4:**
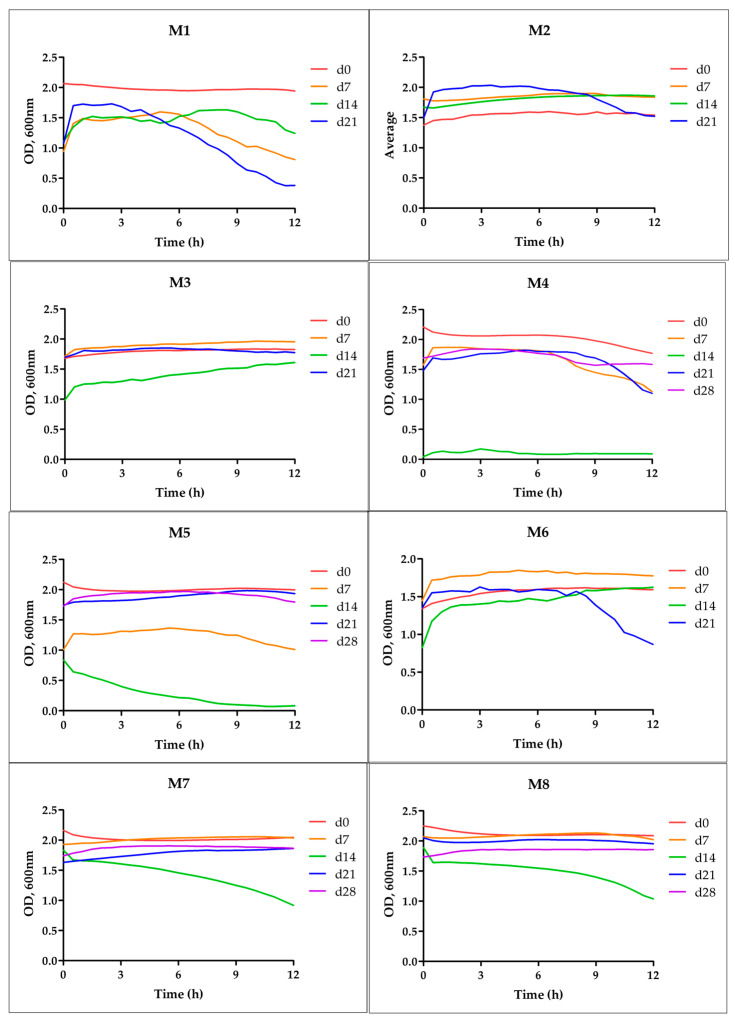
Evolution of physical stability for emulsion formulations M1–M8 over a 28-day storage period. Turbidity was measured as optical density at 600 nm (OD_600nm_) at five time intervals (0, 7, 14, 21, and 28 days). Data are presented as mean (*n* = 3). *Note*: The apparent low OD600 value observed for formulation M4 on day 14 was due to a transient event of partial freezing/sedimentation that affected the specific sample. After resuspension, the sample regained its turbidity.

The freshly prepared emulsions appeared as opaque white suspensions (Figure 3). Time-dependent turbidity profiles (Figure 4) show that most formulations remain stable over 28 days, with only slight declines in optical density (OD 600 nm). Notably, formulations containing PRE (especially M7 and M8) demonstrated the least change in turbidity, suggesting enhanced colloidal stability. This implies that the antioxidant extract may also improve emulsion integrity or steric hindrance. In contrast, ARA-C–only emulsions (M3, M4, M5) showed modest decreases in turbidity over 4 weeks, consistent with some particle coalescence or settling. In polymer-based particulate carriers, aggregation phenomena during prolonged aqueous storage are well documented and motivate the development of stabilization strategies. Several processing strategies have been reported for converting particulate drug systems into dry composites, including vacuum-assisted drying, spray drying, and lyophilization, a well-established strategy to improve long-term stability of polymeric particulate nanomedicine systems [54,55,56].

**Figure 5:**
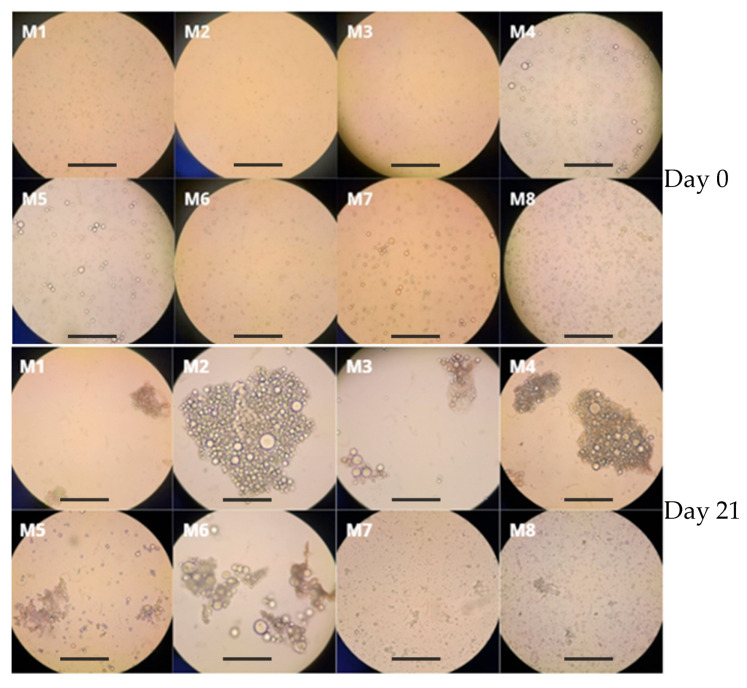
Optical micrographs of formulations M1–M8 showing the initial morphology at day 0 (**top**) and after 21 days of storage at 4 °C (**bottom**). All emulsions exhibit a spherical structure with aqueous droplets encapsulated within the PCL matrix. Scale bars: 100 µm.

Optical micrographs revealed that all formulations produced spherical polymeric particles suspended in the continuous phase (Figure 5). Each particle typically contained aqueous droplets inside the PCL matrix, consistent with the expected W/O/W structure. After 21 days, some aggregates were visible in certain formulations. Notably, M7 and M8 (higher PRE contents) maintained more discrete particles, whereas M2 and M4 (without PRE) showed more clumping.

**Figure 6:**
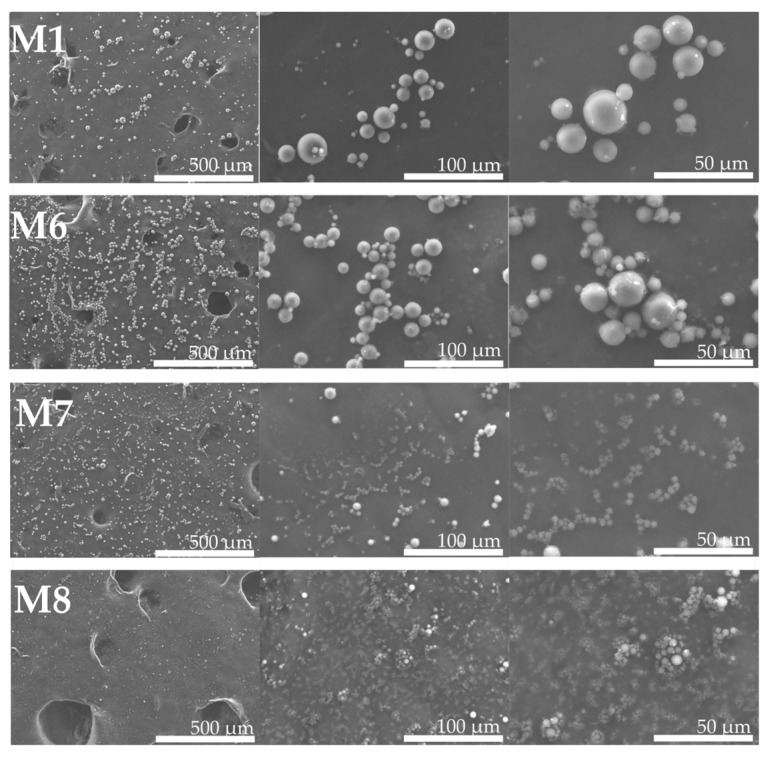
Scanning electron micrographs (SEM) of formulations M1, M6, M7, and M8 after 21 days of storage. Images are presented with scale bars for reference.

SEM imaging confirmed the spherical morphology of the particles. Figure 6 shows representative SEM micrographs for M1, M6, M7, and M8 at various magnifications. All particles appear roughly spherical and smooth, without visible pores. The sizes observed by SEM show a broad size distribution, likely due to the high initial polymer content leading to large droplets. Importantly, M6–M8 (containing both ARA-C and PRE) still formed intact spheres.

DLS analysis provided quantitative size distributions and zeta potentials. The hydrodynamic sizes indicate that the particles of all formulations are in the submicron range (<1 µm). Formulation M1 (no drug) had a mean diameter of 324 ± 214 nm. The co-loaded formulations M6, M7, and M8 had sizes of 450 ± 293, 324 ± 172, and 286 ± 229 nm, respectively (Table 2). Thus, the lowest ARA-C loading (M8) gave the smallest particles (286 nm), whereas higher drug levels (M6: 450 nm) produced larger particles. This suggests that higher hydrophilic content in the inner phase yielded larger emulsion droplets. The sizes are comparable to those reported for other polymeric ARA-C carriers, such as chitosan-ARA-C particles, ~100–300 nm, and PCL/PLA systems, ranging 120–340 nm [57,58]. Zeta potential measurements were conducted at pH 7.4. M1 and M6 were slightly positive (M1: +13.5 mV, M6: +18 mV), whereas M7 and M8 (lower ARA-C) were negative (–35.5 and –42.4 mV). Carboxylic end groups of poly(ε-caprolactone) (pKa ≈ 4–5) contribute a negative background surface charge. Cytarabine is predominantly neutral at physiological pH [59], whereas the major constituents of the polyphenol-rich extract are partially deprotonated at pH 7.4, according to the pKa values reported in the literature, for gallic acid, pKa_1_ = 4.5 (-COOH) [60], and for ellagic acid, pKa_1_ = 6.69 (phenol) [61]. At lower ARA-C loadings, these anionic polyphenols are more likely to be exposed or enriched at the particle–medium interface, leading to strongly negative ζ-potential values. Conversely, higher ARA-C concentrations may partially shield or reorganize surface charges, resulting in near-neutral or slightly positive ζ-potentials.

**Figure 7:**
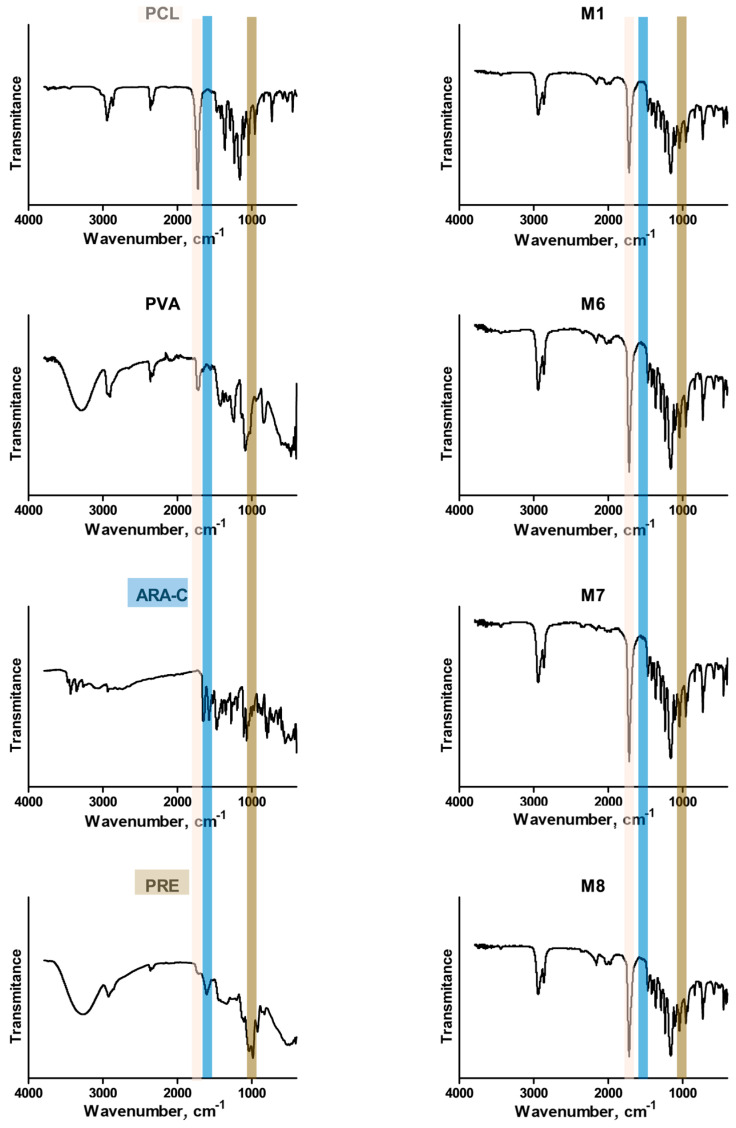
FTIR spectra highlighting characteristic bands of PCL (salmon), ARA-C (blue), and PRE (brown) in the different formulations.

Figure 7 shows the FTIR spectra. The FTIR spectrum of the blank formulation (M1) is dominated by the polycaprolactone fingerprint, a strong ester C=O stretch at ≈1720–1730 cm^−1^. In contrast, the co-loaded formulations (M6–M8) retain these PCL features while exhibiting additional absorptions and pronounced shoulders consistent with both ARA-C and PRE. In this sense, the co-loaded spectra display strengthened signals in the 1650–1600 cm^−1^ window corresponding to heterocyclic C–N vibrations, attributed to ARA-C, and a band at 1030 cm^−1^, corresponding to C–O stretching in the polyphenol skeleton and its derivatives, attributed to the polyphenols in PRE. The spectra were acquired from dried formulations, which rules out strong contributions from solvent bands and supports the interpretation that the observed ARA-C and PRE features are associated with material entrapped inside the microparticles.

Encapsulation efficiency in this study was determined using an indirect method based on quantification of the non-encapsulated fraction in the supernatant, a strategy widely applied for polymeric particulate systems [48,62,63]. Table 2 summarizes the EE results. EE values were consistently high across all formulations, typically exceeding ~80% for both ARA-C and PRE. The high encapsulation agrees with other studies where hydrophilic drugs were effectively loaded into polymeric microparticles. For example, ~64% EE was reported for ARA-C in chitosan nanoparticles [57], whereas our PCL system achieved higher efficiency, possibly due to the different polymer and preparation methods. In general, adding PRE did not diminish ARA-C loading, and vice versa; in fact, some mixed formulations (M8) achieved complete encapsulation of both. Notably, a recent study on PCL-based nanoparticles reported that encapsulation efficiency values obtained by direct (solid-phase analysis) and indirect (supernatant-based UV–Vis quantification) methods were consistent, thereby validating the reliability of the indirect approach [64].

**Figure 8:**
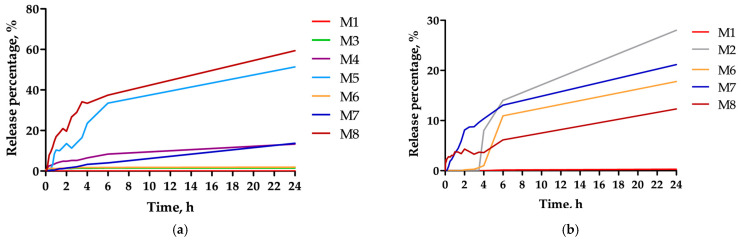
(**a**) *In vitro* release profiles of cytarabine (ARA-C) and (**b**) phenol-rich extract (PRE), from PCL/PVA emulsion matrices in PBS (pH 7.4) (*n* = 3).

A 1 kDa molecular weight cut-off (MWCO) dialysis membrane was selected intentionally for the *in vitro* release experiments because the principal constituents from the pecan polyphenolic extract [24], gallic acid Mw = 170.12 Da [65], ellagic acid Mw = 302.19 Da [61,66], and the chemotherapeutic agent ARA-C, Mw = 243.22 Da [67] all have monomeric molecular weights well below this threshold. Thus, the dialysis system permits free diffusion of these small molecules into the external release medium while retaining intact polymeric microparticles. The *in vitro* release profiles of ARA-C from the microparticles are presented in Figure 8. All formulations (M1–M8) demonstrated significantly attenuated release profiles, effectively mitigating the “burst effect” typical of highly water-soluble drugs. This behavior suggests that the ARA-C molecules are effectively encapsulated within the polymeric matrix, rather than merely adsorbed onto the surface. The release kinetics displayed a characteristic biphasic pattern. First (0–4 h), a moderate release was observed, likely driven by the desorption of drug molecules located at or near the surface. Second, in the sustained phase (4–24 h), the release rate reached a plateau, indicating a transition to a diffusion-controlled mechanism. In comparison to the literature, our ARA-C release is similar. For instance, 70% ARA-C was released in 24 h from chitosan nanoparticles [57], whereas ours required the same period to reach a similar level for the lowest-loaded sample. In contrast, an initial burst of 40% of ARA-C encapsulated in PCL nanoparticles has been reported [58]. However, in that study, poloxamer 188 was used instead of PVA, and the emulsion method was dripping, which may have prevented ARA-C from entering the interior of the particles.

The release from the co-loaded particles was sustained and gradual. Over 24 h, ARA-C cumulative release reached ~70% for the low-loading formulation (M8), whereas higher-loading samples (M3–M6) released 20% in the same period. Therefore, M6 delivers substantially more drug mass. Mechanistically, fractional release represents the proportion of drug released relative to the total loading and does not reflect the absolute mass delivered. Therefore, formulations with higher drug loading may exhibit lower-percentage release while delivering substantially larger drug amounts over the same time period. This apparent discrepancy results from normalization effects, rather than slower release kinetics. PRE release was much slower: even after 24 h, only ~23% of encapsulated polyphenols had diffused out in the highest-release case. Notably, none of the profiles showed an initial burst release, indicating that the drugs were well entrapped, rather than surface-adsorbed. The general trend is that lower drug loading (especially of ARA-C) leads to faster fractional release, consistent with fewer molecules per particle and larger surface area contact. The sustained profiles are expected, given PCL’s low solubility and slow erosion.

Correlation coefficients (R^2^) for release data fitted to common kinetic models are summarized in Table 3 (ARA-C) and Table 4 (PRE). Table 3 shows that the Korsmeyer–Peppas model best describes ARA-C release from formulations M3 and M6, which have the highest concentrations without and with PRE, respectively. Correlation coefficients for PRE release fitted to common kinetic models are summarized in Table 4. The fits indicate formulation-dependent mechanisms, suggesting a more complex release mechanism.

One-way ANOVA on the 24 h ARA-C release percentages showed a significant effect of initial drug concentration (F = 5.12, *p* = 0.007). Post hoc Tukey testing revealed that the release at 5 mg/mL loading was significantly different from that at 0.5 mg/mL, confirming that higher loading retards fractional release. These statistics support the qualitative observation: formulations with lower ARA-C content released proportionally more drug in 48 h.

Pecan kernel extracts exhibit well-documented antioxidant activity *in vitro*, including reactive oxygen species scavenging and inhibition of lipid peroxidation. These effects arise from the combined action of phenolic compounds, which may contribute to protection against oxidative stress, which drives carcinogenic processes [53,68]. Recent reviews emphasize that co-delivery of polyphenols with chemotherapy can enhance therapeutic effect and reduce side effects [69,70,71,72]. Our findings align with this strategy: the sustained co-release of ARA-C and antioxidant extract may allow simultaneous cancer cell kill and ROS mitigation [73]. Compared to reports of polyphenol–chemotherapy nanocarriers, such as resveratrol + docetaxel liposomes [74], our system shows analogous behavior, enhanced stability, and bi-phasic release. Polyphenols exert antioxidant, anti-inflammatory, and cell-signaling modulatory effects and can sensitize cancer cells to chemotherapy. In this case, ARA-C is an S-phase antimetabolite that inhibits DNA synthesis [75]. Co-delivery is, therefore, hypothesized to preserve cytotoxic efficacy via ARA-C, reduce off-target oxidative damage to healthy cells via PRE antioxidants, and potentially enhance therapeutic response in leukemia by modulating survival and stress pathways.

**Figure 9:**
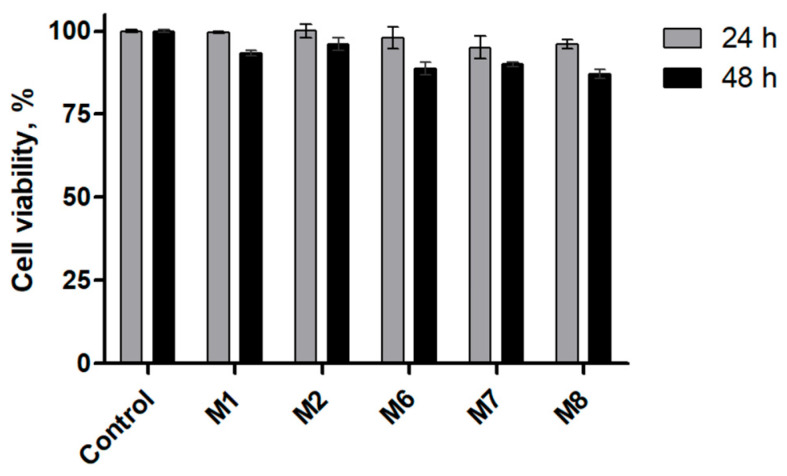
Cell viability of fibroblast subcultures after exposure to PCL-based microparticle formulations, evaluated by the MTT assay at 24 and 48 h. Cell viability is expressed as a percentage relative to untreated controls. Data represent mean ± standard deviation from independent experiments (*n* = 5).

The cytocompatibility of the developed PCL-based microparticles was evaluated using an MTT assay in murine fibroblast subcultures after 24 and 48 h of exposure (Figure 8). This assay was conducted as a preliminary assessment of biocompatibility toward non-malignant cells, an essential requirement for polymeric drug delivery systems intended for biomedical applications. As shown in Figure 9, fibroblasts exposed to blank PCL microparticles (M1) maintained high metabolic activity at both incubation times, with cell viability values close to those of PRE (80 ug/mL) control. This result confirms the intrinsic cytocompatibility of the PCL/PVA matrix, in agreement with previous reports describing PCL as a well-tolerated polymer for drug delivery applications [76,77,78,79]. Formulations containing PRE alone (M2) also preserved high cell viability, consistent with the well-documented antioxidant and cytoprotective properties of plant-derived polyphenols [69,80].

For formulations containing ARA-C, either alone or co-encapsulated with PRE (M3–M8), a moderate reduction in cell viability was observed, particularly at 48 h. This effect is expected, given the antiproliferative nature of cytarabine, even in non-cancerous cells at sufficient concentrations. Importantly, co-loaded formulations (M6–M8) generally exhibited higher fibroblast viability than ARA-C-only formulations at comparable drug loadings, suggesting that the presence of PRE may partially mitigate nonspecific cytotoxic effects. This observation is consistent with the literature reports indicating that polyphenols can reduce oxidative stress and protect normal cells during chemotherapy exposure [81]. The cytotoxicity assays reported here were limited to murine fibroblast subcultures as an initial cytocompatibility screen. Demonstration of specific anti-leukemic activity and potential synergism between ARA-C and PRE will require dedicated studies in leukemia cell lines or primary malignant cells.

## 4. Conclusions

This study reports the development of functional poly(ε-caprolactone)-based microparticles capable of co-encapsulating cytarabine and a pecan-derived polyphenol-rich extract using a double emulsion solvent evaporation method. The system achieved high encapsulation efficiencies; stable colloidal properties; and sustained, formulation-dependent release profiles for both bioactive components. FTIR analysis confirmed the presence of ARA-C and PRE within the polymeric matrix. Preliminary *in vitro* cell viability assays using murine fibroblast subcultures demonstrated that the PCL-based microparticles are cytocompatible under the tested conditions, and that co-encapsulation of polyphenols does not increase nonspecific cytotoxicity associated with cytarabine. These findings support the suitability of the developed polymeric carrier as a nanomedicine platform for combination drug delivery. This work contributes experimental insight into the design of functional polymer systems for the controlled co-delivery of chemotherapeutic agents and natural antioxidants, providing a foundation for future studies focused on antileukemic efficacy in relevant cancer cell models.

## Figures and Tables

**Table 1:** Composition and coding of polycaprolactone microemulsion formulations.

Sample ID	PCL Solution (16 mg/mL) Volume (mL)	PVA Aqueous Phase (2 mg/mL) Volume (mL)	PRE Solution (2.5 mg/mL) Volume (mL)	1.0 mL of ARA-C, Concentration (mg/mL)
M1	5	5	–	–
M2		4	1	–
M3		4	–	50
M4		4	–	5
M5		4	–	0.5
M6		3	1	50
M7		3	1	5
M8		3	1	0.5

**Table 2:** Physicochemical properties and encapsulation efficiency levels of the developed emulsion formulations.

Sample ID	DLS Hydrodynamic Diameter (µm) ± SD	Zeta Potential (mV)	EE ARA-C (%) ± SD	EE PRE (%) ± SD	Remarks
M1	0.324 ± 0.214	+13.5	-	-	Blank control
M2	ND	ND	-	100.00 ± 0.00	PRE only
M3	ND	ND	97.90 ± 0.26	-	ARA-C high
M4	ND	ND	79.56 ± 3.39	-	ARA-C medium
M5	ND	ND	100.00 ± 0.00	-	ARA-C low
M6	0.450 ± 0.293	+18.0	98.10 ± 0.17	98.66 ± 1.89	Co-loaded high
M7	0.324 ± 0.172	−35.5	82.37 ± 0.97	96.94 ± 4.33	Co-loaded medium
M8	0.286 ± 0.229	−42.4	100.00	100.00 ± 0.00	Co-loaded low

ND = not determined. DLS and zeta potential measurements were performed for the co-encapsulated formulations (M6–M8), which were prioritized for physicochemical characterization.

**Table 3:** Correlation coefficients (R^2^) when fitted to kinetic models for the release of ARA-C from emulsions.

Model	Correlation Coefficient (R^2^) *					
	M3	M4	M5	M6	M7	M8
Zero-order	0.2135	0.8139	0.8038	0.3598	0.9189	0.6974
First-order	0.2142	0.8331	0.7957	0.3611	0.9255	0.8447
Higuchi	0.4409	0.9668	0.7181	0.6179	0.5496	0.9133
Hixson	0.24	0.8268	0.7984	0.3607	0.9234	0.7986
Korsmeyer–Peppas	0.8445	0.7959	0.621	0.875	0.5056	0.8118

* R^2^ denotes the coefficient of determination obtained from fitting the experimental release profiles to the corresponding kinetic models

**Table 4:** Correlation coefficients (R^2^) when fitted to kinetic models for the release of PRE from emulsions.

Model	Correlation Coefficient (R^2^)			
	M2	M6	M7	M8
Zero-order	0.9953	0.9846	0.7663	0.862
Fist-order	0.9954	0.9877	0.7952	0.872
Higuchi	0.8833	0.9448	0.8771	0.8855
Hixson	0.9950	0.9868	0.7858	0.8688
Korsmeyer–Peppas	0.3513	0.6269	0.9167	0.6567

## Data Availability

The original contributions presented in this study are included in the article. Further inquiries can be directed to the corresponding authors.

## Abbreviations

The following abbreviations are used in this manuscript:

ALL	Acute lymphoblastic leukemia
ARA-C	Cytarabine
DLS	Dynamic light scattering
DMEM	Dulbecco’s Modified Eagle Medium
DMSO	Dimethyl sulfoxide
EE	Encapsulation efficiency
FTIR	Fourier transform infrared spectroscopy
MTT	3-(4,5-dimethylthiazol-2-yl)-2,5-diphenyltetrazolium bromide
MWCO	Molecular weight cut-off
OD	Optical density
PBS	Phosphate-buffered saline
PCL	Poly(ε-caprolactone)
PRE	Polyphenol-rich extract (from pecan nut)
PVA	Poly(vinyl alcohol)
ROS	Reactive oxygen species
SD	Standard deviation
SEM	Scanning electron microscopy
UV–Vis	Ultraviolet–visible spectroscopy
W/O/W	Water-in-oil-in-water (double emulsion)

## References

[1] Liu W., Fang J., Zhu M., Zhou J., Yuan C. (2025). Global, Regional, and National Burden of Childhood Leukemia from 1990 to 2021. BMC Pediatr..

[2] Bhojwani D., Yang J.J., Pui C.H. (2015). Biology of Childhood Acute Lymphoblastic Leukemia. Pediatr. Clin. N. Am..

[3] Hayashi H., Makimoto A., Yuza Y. (2024). Treatment of Pediatric Acute Lymphoblastic Leukemia: A Historical Perspective. Cancers.

[4] Cheng T., Peng J., Liu Y., Yang S., Chen Y., Xu Y. (2025). Cladribine and Medium-Dose Cytarabine Intensified Busulfan plus Cyclophosphamide Conditioning Regimen for Adults High-Risk B-Cell Acute Lymphoblastic Leukemia. Ann. Hematol..

[5] Cheng L., Zeng S., Yan D., Tu L., Yang Y., Wang X., Zheng X. (2021). Cytarabine and EIP Co-Administration Synergistically Reduces Viability of Acute Lymphoblastic Leukemia Cells with High ERG Expression. Leuk. Res..

[6] Di Francia R., Crisci S., De Monaco A., Cafiero C., Re A., Iaccarino G., De Filippi R., Frigeri F., Corazzelli G., Micera A. (2021). Response and Toxicity to Cytarabine Therapy in Leukemia and Lymphoma: From Dose Puzzle to Pharmacogenomic Biomarkers. Cancers.

[7] Hubeek I., Stam R.W., Peters G.J., Broekhuizen R., Meijerink J.P.P., Van Wering E.R., Gibson B.E.S., Creutzig U., Zwaan C.M., Cloos J. (2005). The Human Equilibrative Nucleoside Transporter 1 Mediates *in vitro* Cytarabine Sensitivity in Childhood Acute Myeloid Leukaemia. Br. J. Cancer.

[8] Wiley J.S., Taupin J., Jamieson G.P., Snook M., Sawyer W.H., Finch L.R. (1985). Cytosine Arabinoside Transport and Metabolism in Acute Leukemias and T Cell Lymphoblastic Lymphoma. J. Clin. Investig..

[9] Shen Y., Li Y., Yan R. (2024). Structural Basis for the Inhibition Mechanism of the DNA Polymerase Holoenzyme from Mpox Virus. Structure.

[10] Dalisay D.S., Tenebro C.P., Sabido E.M., Suarez A.F.L., Paderog M.J.V., Reyes-Salarda R., Saludes J.P. (2024). Marine-Derived Anticancer Agents Targeting Apoptotic Pathways: Exploring the Depths for Novel Cancer Therapies. Mar. Drugs.

[11] Hamada A., Kawaguchi T., Nakano M. (2002). Clinical Pharmacokinetics of Cytarabine Formulations. Clin. Pharmacokinet..

[12] Martel A.L., Fraleigh N.L., Picard E., Lewicky J.D., Pawelec G., Lee H., Ma G.W., Mousavifar L., Roy R., Le H.T. (2021). Novel Immunomodulatory Properties of Low Dose Cytarabine Entrapped in a Mannosylated Cationic Liposome. Int. J. Pharm..

[13] Yu Y.M., Bu F.Z., Meng S.S., Yan C.W., Wu Z.Y., Li Y.T. (2023). The First Nano-Cocrystal Formulation of Marine Drug Cytarabine with Uracil Based on Cocrystal Nanonization Strategy for Long-Acting Injection Exhibiting Enhanced Antitumor Activity. Int. J. Pharm..

[14] Kegyes D., Moisoiu V., Constantinescu C., Tanase A., Ghiaur G., Einsele H., Tomuleasa C., Lazarus H.M., Gale R.P. (2025). Neuro-Toxicities of Chemo- and Immune-Therapies in Haematologic Malignancies: From Mechanism to Management. Blood Rev..

[15] Jakobušić Brala C., Karković Marković A., Kugić A., Torić J., Barbarić M. (2023). Combination Chemotherapy with Selected Polyphenols in Preclinical and Clinical Studies—An Update Overview. Molecules.

[16] Chapa González C., Stevens Barrón J.C. (2024). Los Beneficios Potenciales de Los Extractos de Frutos Secos En El Tratamiento Del Cáncer. Cienc. Vital.

[17] Oriol-Caballo M., Moreno-Murciano M.P., López-Blanch R., Estrela J.M., Obrador E. (2025). Polyphenols: Potential Applications in Cancer Therapy. Mol. Nutr. Food Res..

[18] Islam M.A., Medha M.M., Nahar A.U., Al Fahad M.A., Siraj M.A., Seidel V. (2023). Cancer Protective Role of Selected Dietary Polyphenols via Modulating Keap1/Nrf2/ARE and Interconnected Signaling Pathways. Nutr. Cancer.

[19] Guan C., Zhou X., Li H., Ma X., Zhuang J. (2022). NF-ΚB Inhibitors Gifted by Nature: The Anticancer Promise of Polyphenol Compounds. Biomed. Pharmacother..

[20] Mileo A.M., Miccadei S. (2015). Polyphenols as Modulator of Oxidative Stress in Cancer Disease: New Therapeutic Strategies. Oxid. Med. Cell. Longev..

[21] de La Rosa L.A., Alvarez-Parrilla E., Shahidi F. (2011). Phenolic Compounds and Antioxidant Activity of Kernels and Shells of Mexican Pecan (*Carya illinoinensis*). J. Agric. Food Chem..

[22] de la Rosa L.A., Álvarez-Parrilla E., García-Fajardo J.A. (2019). Identificación de Compuestos Fenólicos En Extractos de Almendra (*Prunus dulcis*) y Nuez Pecana (*Carya illinoinensis*) Mediante Cromatografía Líquida Acoplada a Espectrometría de Masas En Tándem (HPLC-MS/MS). TIP Rev. Espec. Cienc. Químico-Biológicas.

[23] Álvarez-Parrilla E., Urrea-López R., de la Rosa L.A. (2018). Bioactive Components and Health Effects of Pecan Nuts and Their By-Products: A Review. J. Food Bioact..

[24] Reyes-Vázquez N., de la Rosa L.A., Morales-Landa J.L., García-Fajardo J.A., García-Cruz M.Á. (2022). Phytochemical Content and Potential Health Applications of Pecan [*Carya illinoinensis* (Wangenh) K. Koch] Nutshell. Curr. Top. Med. Chem..

[25] Sen C.K., Khanna S., Roy S. (2006). Tocotrienols: Vitamin E Beyond Tocopherols. Life Sci..

[26] Abraham A., Kattoor A.J., Saldeen T., Mehta J.L. (2019). Vitamin E and Its Anticancer Effects. Crit. Rev. Food Sci. Nutr..

[27] Dasgupta J., Sanyal U., Das S. (1993). Vitamin E--Its Status and Role in Leukemia and Lymphoma. Neoplasma.

[28] Morgan N.R., Ramdas P., Bhuvanendran S., Radhakrishnan A.K. (2024). Delineating the Immunotherapeutic Potential of Vitamin E and Its Analogues in Cancer: A Comprehensive Narrative Review. Biomed Res. Int..

[29] Mateș L., Banc R., Zaharie F.A., Rusu M.E., Popa D.S. (2024). Mechanistic Insights into the Biological Effects and Antioxidant Activity of Walnut (*Juglans regia* L.) Ellagitannins: A Systematic Review. Antioxidants.

[30] Stevens-Barrón J.C., Wall-Medrano A., Álvarez-Parrilla E., Olivas-Armendáriz I., Astiazaran-García H., Robles-Zepeda R.E., De la Rosa L.A. (2022). Synergistic Interactions between Tocol and Phenolic Extracts from Different Tree Nut Species against Human Cancer Cell Lines. Molecules.

[31] Ribas L.E., Baravalle M.E., Gasser F.B., Renna M.S., Addona S., Ortega H.H., Savino G.H., Van de Velde F., Hein G.J. (2021). Extraction of Phenolic Compounds from the Shells of Pecan Nuts with Cytotoxic Activity through Apoptosis against the Colon Cancer Cell Line HT-29. J. Food Sci..

[32] Sahu K.L., Naeem K., Carson M.R., Nawaz N.K., Gulfam N., Zahoor M. (2025). Advancements in Polycaprolactone Nanoparticles for Targeted Drug Delivery in Breast Cancer Treatment: Strategies and Challenges. Next Nanotechnol..

[33] Greatti V.R., Oda F., Sorrechia R., Kapp B.R., Seraphim C.M., Weckwerth A.C.V.B., Chorilli M., Da Silva P.B., Eloy J.O., Kogan M.J. (2020). Poly-ε-Caprolactone Nanoparticles Loaded with 4-Nerolidylcatechol (4-NC) for Growth Inhibition of Microsporum Canis. Antibiotics.

[34] Aguiar A., Loureiro M.V., Pinho I., Marques A.C. (2023). Efficient Encapsulation of Isocyanates in PCL/PLA Biodegradable Microcapsules for Adhesives. J. Mater. Sci..

[35] Gurler E.B., Ergul N.M., Ozbek B., Ekren N., Oktar F.N., Haskoylu M.E., Oner E.T., Eroglu M.S., Ozbeyli D., Korkut V. (2019). Encapsulated Melatonin in Polycaprolactone (PCL) Microparticles as a Promising Graft Material. Mater. Sci. Eng. C.

[36] Procopio A., Lagreca E., Jamaledin R., La Manna S., Corrado B., Di Natale C., Onesto V. (2022). Recent Fabrication Methods to Produce Polymer-Based Drug Delivery Matrices (Experimental and In Silico Approaches). Pharmaceutics.

[37] Mashhadian A., Afjoul H., Shamloo A. (2022). An Integrative Method to Increase the Reliability of Conventional Double Emulsion Method. Anal. Chim. Acta.

[38] Li D., Zhao Y., Zhang Y., An J., Huang J., Yang J. (2024). Encapsulation of Hydrophobic-but-Not-Lipophilic Perfluoro Liquids Based on a Self-Assembled Double Emulsion Template via Solvent Evaporation Method. ACS Appl. Mater. Interfaces.

[39] Safari H., Felder M.L., Kaczorowski N., Eniola-Adefeso O. (2022). Effect of the Emulsion Solvent Evaporation Technique Cosolvent Choice on the Loading Efficiency and Release Profile of Anti-CD47 from PLGA Nanospheres. J. Pharm. Sci..

[40] de Almeida Campos L.A., de Souza J.B., de Queiroz Macêdo H.L.R., Borges J.C., de Oliveira D.N., Cavalcanti I.M.F. (2024). Synthesis of Polymeric Nanoparticles by Double Emulsion and PH-Driven: Encapsulation of Antibiotics and Natural Products for Combating *Escherichia coli* Infections. Appl. Microbiol. Biotechnol..

[41] Benavides Castillo L., Martinez Y. (2023). The Concentration and Type of Emulsifier Rules the Oil/Water and Water/Oil/Water Emulsion Size Distribution. Chem. Eng. Commun..

[42] Bakouei M., Kalantarifard A., Sundara Raju I., Avsievich T., Rannaste L., Kreivi M., Elbuken C. (2024). Facile and Versatile PDMS-Glass Capillary Double Emulsion Formation Device Coupled with Rapid Purification toward Microfluidic Giant Liposome Generation. Microsyst. Nanoeng..

[43] Vaida C., Mela P., Kunna K., Sternberg K., Keul H., Möller M. (2010). Microparticles for Drug Delivery Based on Functional Polycaprolactones with Enhanced Degradability: Loading of Hydrophilic and Hydrophobic Active Compoundsa. Macromol. Biosci..

[44] Bhadran A., Shah T., Babanyinah G.K., Polara H., Taslimy S., Biewer M.C., Stefan M.C. (2023). Recent Advances in Polycaprolactones for Anticancer Drug Delivery. Pharmaceutics.

[45] Sinha V.R., Bansal K., Kaushik R., Kumria R., Trehan A. (2004). Poly-ϵ-Caprolactone Microspheres and Nanospheres: An Overview. Int. J. Pharm..

[46] Terrazas García D.F., de la Rosa L.A., Vázquez Flores A.A., Muñoz Bernal O.A., Stevens Barrón J.C., Chapa González C. (2025). Nanostructured Delivery Systems for Antioxidants: Comparative Release of Purified Ellagic Acid and Extracted Polyphenols. Nano-Struct. Nano-Objects.

[47] Amini Y., Amel Jamehdar S., Sadri K., Zare S., Musavi D., Tafaghodi M. (2017). Different Methods to Determine the Encapsulation Efficiency of Protein in PLGA Nanoparticles. Biomed. Mater. Eng..

[48] Daneshmand S., Golmohammadzadeh S., Jaafari M.R., Movaffagh J., Rezaee M., Sahebkar A., Malaekeh-Nikouei B. (2018). Encapsulation Challenges, the Substantial Issue in Solid Lipid Nanoparticles Characterization. J. Cell. Biochem..

[49] Muso-Cachumba J.J., Ruiz-Lara G., Monteiro G., Rangel-Yagui C.d.O. (2023). Challenges in Estimating the Encapsulation Efficiency of Proteins in Polymersomes—Which Is the Best Method?. Braz. J. Pharm. Sci..

[50] Martinez-Osuna D., Olivas-Armendariz I., Estrada-Rojas P., Jimenez-Vega F., Mendoza-Duarte M.E., Vega-Rios A., Chapa-Gonzalez C., Martel-Estrada S.-A., Valencia-Gomez L.E., Salcedo M. (2025). Silver Sulfide Quantum Dots Conjugated with Anti-PSG1 Monoclonal Antibodies: Optical, Photothermal, and Cytocompatibility Assessment. Processes.

[51] do Prado A.C.P., da Silva H.S., da Silveira S.M., Barreto P.L.M., Vieira C.R.W., Maraschin M., Ferreira S.R.S., Block J.M. (2014). Effect of the Extraction Process on the Phenolic Compounds Profile and the Antioxidant and Antimicrobial Activity of Extracts of Pecan Nut [*Carya illinoinensis* (Wangenh) C. Koch] Shell. Ind. Crops Prod..

[52] Villarreal-Lozoya J.E., Lombardini L., Cisneros-Zevallos L. (2007). Phytochemical Constituents and Antioxidant Capacity of Different Pecan [*Carya illinoinensis* (Wangenh.) K. Koch] Cultivars. Food Chem..

[53] de la Rosa L.A., Vázquez-Flores A.A., Álvarez-Parrilla E., Rodrigo-García J., Medina-Campos O.N., Ávila-Nava A., González-Reyes S., Pedraza-Chaverri J. (2014). Content of Major Classes of Polyphenolic Compounds, Antioxidant, Antiproliferative, and Cell Protective Activity of Pecan Crude Extracts and Their Fractions. J. Funct. Foods.

[54] Gatto M.S., Najahi-Missaoui W. (2023). Lyophilization of Nanoparticles, Does It Really Work? Overview of the Current Status and Challenges. Int. J. Mol. Sci..

[55] Ojha T., Hu Q., Colombo C., Wit J., van Geijn M., van Steenbergen M.J., Bagheri M., Königs-Werner H., Buhl E.M., Bansal R. (2021). Lyophilization Stabilizes Clinical-Stage Core-Crosslinked Polymeric Micelles to Overcome Cold Chain Supply Challenges. Biotechnol. J..

[56] Fonte P., Reis S., Sarmento B. (2016). Facts and Evidences on the Lyophilization of Polymeric Nanoparticles for Drug Delivery. J. Control. Release.

[57] Deepa G., Sivakumar K.C., Sajeevan T.P. (2018). Molecular Simulation and *in vitro* Evaluation of Chitosan Nanoparticles as Drug Delivery Systems for the Controlled Release of Anticancer Drug Cytarabine against Solid Tumours. 3 Biotech.

[58] Jan N., Madni A., Rahim M.A., Khan N.U., Jamshaid T., Khan A., Jabar A., Khan S., Shah H. (2021). *In vitro* Anti-Leukemic Assessment and Sustained Release Behaviour of Cytarabine Loaded Biodegradable Polymer Based Nanoparticles. Life Sci..

[59] Cytarabine: Uses, Interactions, Mechanism of Action|DrugBank. https://go.drugbank.com/drugs/DB00987.

[60] Molski M. (2025). Computation of the PKa Values of Gallic Acid and Its Anionic Forms in Aqueous Solution: A Self-Similar Transformation Approach for Accurate Proton Hydration Free Energy Estimation. Molecules.

[61] Wang W., Wang S., Liu Y., Wang X., Nie J., Meng X., Zhang Y. (2022). Ellagic Acid: A Dietary-Derived Phenolic Compound for Drug Discovery in Mild Cognitive Impairment. Front. Aging Neurosci..

[62] Wallace S.J., Li J., Nation R.L., Boyd B.J. (2012). Drug Release from Nanomedicines: Selection of Appropriate Encapsulation and Release Methodology. Drug Deliv. Transl. Res..

[63] Baltz N., Scherließ R. (2025). Entrapment Efficiency Methodology for Lipid Nanoparticles—A Literature Review. OpenNano.

[64] Massella D., Celasco E., Salaün F., Ferri A., Barresi A.A. (2018). Overcoming the Limits of Flash Nanoprecipitation: Effective Loading of Hydrophilic Drug into Polymeric Nanoparticles with Controlled Structure. Polymers.

[65] Hadidi M., Liñán-Atero R., Tarahi M., Christodoulou M.C., Aghababaei F. (2024). The Potential Health Benefits of Gallic Acid: Therapeutic and Food Applications. Antioxidants.

[66] Golmei P., Kasna S., Roy K.P., Kumar S. (2024). A Review on Pharmacological Advancement of Ellagic Acid. J. Pharmacol. Pharmacother..

[67] Arwanih E.Y., Louisa M., Rinaldi I., Wanandi S.I. (2022). Resistance Mechanism of Acute Myeloid Leukemia Cells Against Daunorubicin and Cytarabine: A Literature Review. Cureus.

[68] Ezeanolue I.R., Ezeanolue C.F., Plastina P., Stefanello F.M., Giacomelli Tavares R., Spanevello R.M. (2025). *In vitro* Anti-Inflammatory and Anticancer Potential of Pecan Nut (*Carya illinoinensis*) Kernel Extracts: Modulation of Cell Signaling Pathways—A Scoping Review. Molecules.

[69] Hamdy N.M., Amin A., Abd-ellatef G.E.F., Abdalla Y., Abdalla A., Saqr D.A., Lu Y., Wu W., Gamal El-Din M.I., El-Shazly M. (2026). An Overview of Targeting Some Cancer Hallmarks with Plant Polyphenols: A Step Toward Precision.

[70] Bolaños-Cardet J., Pepió-Tárrega B., Saiz-Poseu J., López-Moral A., Ullah F., Yuste V.J., Ruiz-Molina D., Suárez-García S. (2025). The Redox Properties of Polyphenols and Their Role in ROS Generation for Biomedical Applications. Angew. Chem.—Int. Ed..

[71] Vieira I.R.S., Tessaro L., Lima A.K.O., Velloso I.P.S., Conte-Junior C.A. (2023). Recent Progress in Nanotechnology Improving the Therapeutic Potential of Polyphenols for Cancer. Nutrients.

[72] Vladu A.F., Ficai D., Ene A.G., Ficai A. (2022). Combination Therapy Using Polyphenols: An Efficient Way to Improve Antitumoral Activity and Reduce Resistance. Int. J. Mol. Sci..

[73] Adeyemi S.A., Ngema L.M., Choonara Y.E. (2025). Advances in Targeted Therapies and Emerging Strategies for Blood Cancer Treatment. RSC Pharm..

[74] Zhang L., Lin Z., Chen Y., Gao D., Wang P., Lin Y., Wang Y., Wang F., Han Y., Yuan H. (2022). Co-Delivery of Docetaxel and Resveratrol by Liposomes Synergistically Boosts Antitumor Efficiency against Prostate Cancer. Eur. J. Pharm. Sci..

[75] Momparler R.L. (2013). Optimization of Cytarabine (ARA-C) Therapy for Acute Myeloid Leukemia. Exp. Hematol. Oncol..

[76] Boyuklieva R., Hristozova A., Pilicheva B. (2023). Synthesis and Characterization of PCL-Idebenone Nanoparticles for Potential Nose-to-Brain Delivery. Biomedicines.

[77] Rahmani D., Torbat N.A., Boddohi S. (2023). Synthesis and Characterization of PH-Responsive PCL-PVA Polymersome for Dual Delivery to Breast Cancer Cells. Eur. Polym. J..

[78] Grossen P., Witzigmann D., Sieber S., Huwyler J. (2017). PEG-PCL-Based Nanomedicines: A Biodegradable Drug Delivery System and Its Application. J. Control. Release.

[79] Valdez Medina K.A., Medina Salas G.A., Morales Jacquez S.A., Carrillo Castillo A., Chapa González C., Illescas Martínez F.J., Pugh C., Duhamel J., González Méndez I. (2024). Development of PCL Nanofibers for Delivery of Pecan Bioactive Compounds in Adjunctive Leukemia Treatment. Proceedings of the 32IMRC. E6. Polymers as Versatile Materials: Design, Preparation, Characterization, Properties, and Extended Applications, Cancún, México, 18–23 August 2024.

[80] Maruszewska A., Tarasiuk J. (2019). Antitumour Effects of Selected Plant Polyphenols, Gallic Acid and Ellagic Acid, on Sensitive and Multidrug-Resistant Leukaemia HL60 Cells. Phytother. Res..

[81] Fakhar F., Mohammadian K., Keramat S., Stanek A. (2024). The Potential Role of Dietary Polyphenols in the Prevention and Treatment of Acute Leukemia. Nutrients.

